# Address of President Stewart before the Missouri Valley Veterinary Association

**Published:** 1896-08

**Authors:** 


**On to Buffalo!**

---

### ADDRESS OF PRESIDENT STEWART AT THE ANNUAL MEETING OF THE MISSOURI VALLEY VETERINARY ASSOCIATION, JUNE, 1896.

THIS meeting completes the second year of our organization, and we are pleased to think that the energy put forth in the eight quarterly sessions has redounded to the advantage of our members and has increased our professional interests in many ways. Each meeting has been fruitful in practical, helpful essays and reports; we have become better acquainted each with the other; we have grown to a better understanding of professional ethics, and have striven for a fuller application of their most wholesome precepts. It is very gratifying to note the general kindliness which has characterized the interchange of thought in our discussions and the charity with which individual hobbies have been received. I sincerely trust this fraternal spirit will continue to grow in strength and shed its beneficent influence over this Association. It is indeed a great pleasure to be able to say to you that it has been a long time since an unkind or uncharitable word has been said in my presence concerning a member of this body, or, for that matter, concerning members of our profession anywhere.

Our membership is sufficient to maintain an active, aggressive organization, and we will discredit our opportunities if we fail to cultivate and disseminate a knowledge of the veterinarian and his capacity to protect the public health, as well as prevent pain and sickness in our domestic animals while conserving their financial value.

The public knows in a general way that the national government has established a sanitary inspection of meats in our large slaughtering establishments, and "thinks it a good thing," but it does not seem to realize the necessity of inspection of the animals killed at the small or local slaughter-houses, and, yet more important, the inspection of the dairy and its products. It is properly within our duty to keep this subject before our respective communities and do what we can to awaken public interest along this line.

These are times of wonderful growth in veterinary science, and the thought often arises, Are we keeping up with the advance or are we retrograding? Are we gleaning with interest and eagerness the increasing knowledge and ripening thought along its many divisions? We are probably fairly awake on the subject of tuberculosis, because it has been discussed in every meeting of veterinarians held during the last several years. Are our ideas clear on other subjects of great importance? Dr. Parker's recent contribution on "Milk as a Factor in the Causation of Disease" is worthy of very careful reading, and leads to the far-reaching problem of cleanliness in the handling and preparation of all foods, and indicates that future meat- and milk-inspection must include possible contamination from extraneous sources as well as by animals afflicted with disease. It is positively necessary for us to read the current veterinary and medical literature if we keep apace; and I trust none have found money so difficult to get that he has not secured one, if not all, of our veterinary journals which are full to overflowing with the newest and best thoughts of our profession. Through their columns we learn something of the magnitude of the efforts being made looking toward the control and probable eradication of bovine tuberculosis in several States of this nation and in some European countries, and we must note the large number of veterinarians employed in this work. It is largely through our journals that we become acquainted with new remedies and new uses of old ones; that we are apprised of the advent of new diseases and the more successful treatment of old ones. How few of us feel that we have made as comprehensive and practical an application of asepsis and antisepsis in our daily practice as we might have done? I trust some member can report on the value and application of campho-phenique, hydrozone, trikresol, etc.; can report successful cases of healing without suppuration in castration and spaying, neurectomy and ablation of tumors, and other varieties of surgical cases. Is there any good reason why veterinarians should be content with old methods and results? With reasonable skill and modern methods a copious flow of laudable pus should not be looked upon as a gratifying and desirable state in the healing process following surgical operations.

The wondrous development of serum-therapy is of such recent date and its possibilities so great that one is benumbed with amazement and incredulity when the facts are presented, if he has not read the journals. What with tuberculin and mallein, antitoxin for diphtheria and tetanus, vaccines for anthrax and hog cholera, etc., and, last of all, the application of electricity in the form of the x-rays for the destruction of pathogenic microbes and the diagnosis of fractures or the presence of tumors and foreign bodies hidden in the deep tissues, who is able to foresee what the future medical science will be; who can even fully comprehend its present state?

Association tends to quicken our perceptions and stimulates our zeal, and I feel sure we are encouraged with the hope of yet better and more certain results in the treatment of our patients and the preservation of the property of our clients by the intelligent use of the new agencies at our command.

We must certainly be grateful for the opportunity which our professional education opens for us to promote the public weal, and should be ever alert to help forward the movement so well begun in several States.

As witness of the progress being made you will recall the legislative enactments of several States, and the hopeful prospect of the army veterinary bill. A half-dozen energetic veterinarians in Virginia have secured a substantial law requiring a State certificate to entitle a veterinarian to practice, and are confident they will secure another law requiring and controlling dairy-inspection. Pennsylvania, New York, Ohio, North Dakota, and California have made laws governing practice. Several cities are enforcing quite rigid veterinary inspection of markets and dairies. Are we cultivating public opinion in our several communities? It is fair to presume that the people of Missouri and Kansas are just as anxious to ward off all diseases and protect themselves from financial loss through veterinary pretenders and sharks as other States, if they were aware of the sources of such dangers and losses. It remains for us to impart such information.

We are entitled to be proud of our profession, and I trust each one of us may add his mite toward its uplifting and its progress.

